# *Sphenostylis stenocarpa* (ex. A. Rich.) Harms., a Fading Genetic Resource in a Changing Climate: Prerequisite for Conservation and Sustainability

**DOI:** 10.3390/plants6030030

**Published:** 2017-07-12

**Authors:** Catherine Veronica Nnamani, Sunday Adesola Ajayi, Happiness Ogba Oselebe, Christopher John Atkinson, Anastasia Ngozi Igboabuchi, Eucharia Chizoba Ezigbo

**Affiliations:** 1 Plant Taxonomy/Biosystematics and Conservation Biology Research Lab, Department of Applied Biology, Ebonyi State University, Abakaliki 840001, Nigeria; chyzonroyal@gmail.com; 2 Department of Crop Production and Protection, Obafemi Awolowo University, Ile-Ife 220282, Nigeria; sajayi@oauife.edu.ng; 3 Department of Crop Production & Landscape Management, Ebonyi State University, Abakaliki 840001, Nigeria; happinessoselebe@yahoo.com; 4 Natural Resources Institute, University of Greenwich, Medway Campus, Central Avenue, Chatham Maritime, Kent ME4 4TB, UK; c.j.atkinson@greenwich.ac.uk; 5 Department of Biology, Nwafor Orizu College of Education, Nsugbe 240001, Nigeria; mumngozi@gmail.com

**Keywords:** African Yam Bean, indigenous knowledge, genetic erosion, conservation, food security, Nigeria

## Abstract

The southeastern part of Nigeria is one of the major hotspots of useful plant genetic resources. These endemic species are associated with a rich indigenous knowledge and cultural diversity in relation to their use and conservation. *Sphenostylis stenocarpa* (*e*x. A. Rich.) Harms., (African Yam Bean (AYB)), is one such crop within the family of Fabaceae. Its nutritional and eco-friendly characteristics have value in ameliorating malnutrition, hidden hunger and environmental degradation inherent in resource-poor rural and semi-rural communities throughout Africa. However, lack of information from the custodians of this crop is limiting its sustainable development. Therefore, ethnobotanical surveys on the diversity, uses, and constraints limiting the cultivation and use of the crop in southeastern Nigeria were carried out. Five-hundred respondents were randomly selected and data collected through oral interviews and focused group discussion (FGD). Semi-structured questionnaires (SSQ) were also used to elicit information from a spectrum of AYB users comprising community leaders, farmers, market women and consumers in five States. Results showed that the majority of the respondents lacked formal education and were of the age group of 40–50 years, while the female gender dominated with limited access to land and extension officers. Seed coat colour largely determined utilization. Long cooking time, requirement for staking materials, aging of farmers and low market demand were among the major constraints limiting further cultivation and utilization of AYB. In-situ conservation was by hanging dried fruits by the fireside, beside the house, storing in earthenware, calabash gourds, cans and bottles. It is concluded that there is urgent need to scale up conservation through robust linkages between contemporary scientific domains and indigenous peoples in order to harness and incorporate the rich indigenous knowledge in local communities for enhanced scientific knowledge, biodiversity conservation and its sustainable utilization for food security.

## 1. Introduction

The dependence of humans on plants for their livelihood is connected to the development of specific knowledge on plant value, use, management, and conservation [1,2]. The Convention on Biological Diversity [3] stresses the need to respect, preserve and maintain the knowledge, innovation and practices of indigenous communities relevant for the conservation and sustainable use of biological diversity. The direct relationship between biological and cultural diversity shows that the maintenance of the former can help preserve the latter while increasing the capacity of human to adapt to change [4]. Just as biological diversity underpins the resilience of natural systems, so does cultural diversity [5] and this increases the resilience of social systems. Indigenous people are the major custodians of knowledge on endemic biodiversity because of the long and intertwined associations between their survival and the utilization of plant species for food, traditional medicine and a diversity of other uses. Harnessing this information can strengthen research in the contemporary scientific domain on AYB.

Ethnobotanical information is essential for assessing both the diversity and the adaptation characteristics of useful plants and this helps in understanding a plants’ micro-niches and the stability of environmental conditions. It is also useful in the collection of the genetic resources of these cultivated and economically important species in order to capture variation within these species [6]. Ethnobotany serves as an untapped reservoir of knowledge, especially with respect to the interactions between plants, people, folk taxonomy, plant mythology, ethnomedicine, food security, environment restoration and germplasm conservation. Unfortunately, the indigenous knowledge systems are fast eroding due to several anthropogenic factors such as colonialism, commercialization, globalization, modernization, breakdown of the African traditional family structures, developmentally-induced human displacements and urban migration, all of which have induced a lack of interest from the younger generation [7].

Nigeria is one of the hotspots for plants genetic resources and cultural diversity [8,9]. Hence, the various geographical zones provide a platform for the study of perceptions of the custodians of indigenous knowledge about plant genetic resources.

*Sphenostylis stenocarpa* (*e*x. A. Rich.) Harms, commonly known as African yam bean (AYB), is a neglected and underutilized leguminous plant genetic resource of the subfamily Faboideae, family Fabaceae and a small genus represented by only seven species [10]. It is a perennial climbing species whose morphotypes may also be prostrate, or erect and about 1–3 m in height. Its leaves are trifoliate, 2.7 to 13 cm long and 0.2 to 5.5 cm broad. The inflorescence is a raceme that exhibits an acropetal mode of floral maturation with pink flowers blended with purple, with the slightly twisted backward characteristic of the Fabaceae [11]. The center of diversity of AYB is the northeast tropical Africa (Chad and Ethiopia), east tropical Africa (Kenya, Tanzania and Uganda), west-central tropical Africa (Burundi, Central African Republic and Zaire), West Africa (Cote d’Ivoire, Ghana, Guinea, Mali, Niger, Nigeria and Togo) and south tropical Africa (Angola, Malawi, Zambia and Zimbabwe) [12,13].

African yam bean is cultivated for its edible tubers and for its seeds which have high nutritional values. The amino acid (lysine and methionine) has been reported to be higher than those of pigeon pea, cowpea, and bambara groundnut [14]. Omeire [15], noted that the amino acid (g/100 g) profile of African yam bean comprised lysine (6.12), histidine (3.10), arginine (6.47), aspartic acid (9.12), glycine (3.90), alanine (4.05), valine (4.96), and phenylalanine (5.05). Its lysine and methionine contents were equal to or better than those of soybean protein [16], while equally comparable with whole chicken eggs and can meet the daily human requirement for protein [17]. Its protein profile compares favourably with that of other African root crops such as yams, sweet potatoes and has almost ten times the protein value of cassava tubers, while the essential proteins in AYB are similar to those in soybeans [18].

In spite of the extensive information on the morphological characterization for yield [11,13,19,20], biochemical profiles [21,22,23], physiological seed quality [24], and genetic diversity [25,26,27,28] of AYB, there is dearth of information on ethnobotanical knowledge on this highly-promising plant genetic resource.

Therefore, the aim of this study was to carry out an ethnobotanical survey to develop a hub of indigenous knowledge on AYB and to complement this with the existing wealth of scientific knowledge. This study sought to systematically bridge the knowledge gap by (a) identifying the socio-economic characteristics of respondents involved in AYB activities in five states of southeastern Nigeria; (b) determine the uses of AYB and phenotypic variability in seed coat colour and (c) identify those constraints limiting the use and production systems of AYB in southeastern Nigeria.

## 2. Results and Discussion

### 2.1. Social Background of Respondents

**Figure 1:**
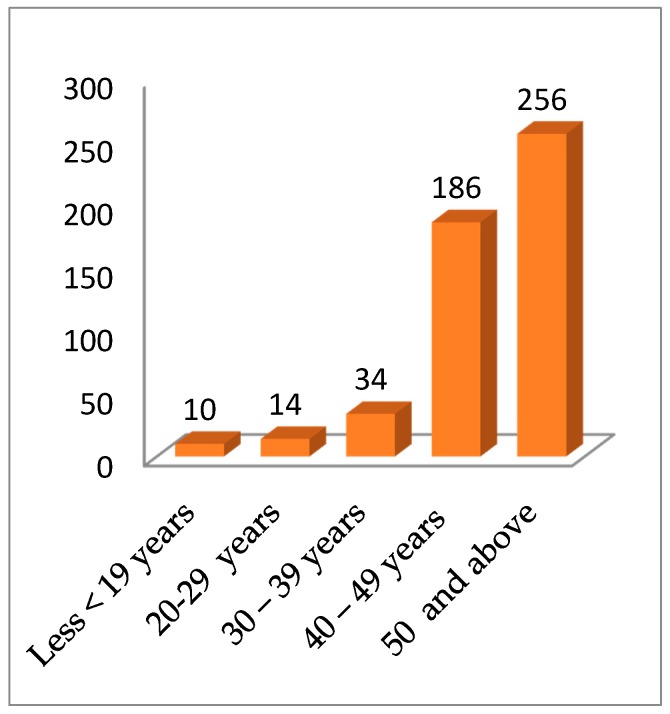
Age range of respondents from the five states of southeastern, Nigeria.

**Figure 2:**
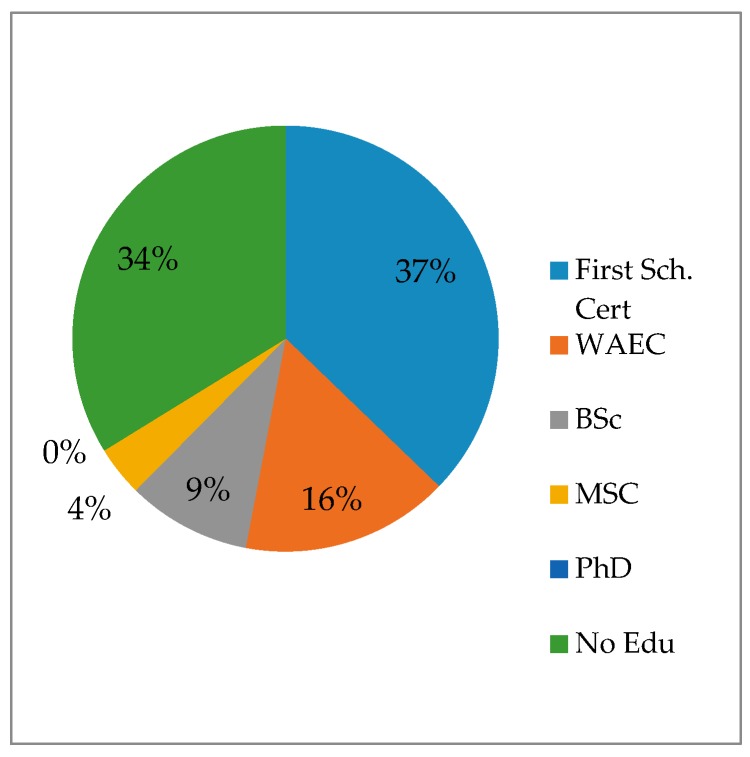
Educational background status of respondents from the five states of South eastern, Nigeria.

Two-hundred-fifty-six (51.2%) of the 500 respondents were 50 years old and were involved either in the cultivation and/or sales of AYB products as a source of food and income. Two percent (2%), 2.5% and 6.8% of the respondents were within the age limits of <19, 20–29 and 30–39, respectively (Figure 1). With respect to the level of education 37.2% had primary school-leaving certificates while 34% had no formal education (Figure 2). These results are similar to those of Mathibela et al. [29], who reported that 64% of traditional healers, who were the custodians of indigenous knowledge in medicinal plants in the Blouberg area of South Africa, had no formal education, while 32% had primary school certificates, with only 4% having attended secondary school. The implications of these results are that potentially valuable information on these plants is in the hands of the older generation who by virtue of their age have a diminishing involvement in AYB cultivation. Their educational levels may not limit them from documenting this knowledge for posterity and for the benefit of people outside the local community. With the very low involvement of younger people in farming in general and specifically in the cultivation of AYB, it is reasonable to assume that there will be a progressive decline and eventually a substantial loss of indigenous AYB cultural knowledge and genetic diversity over temporal and spatial scales. This is in line with the opinion of Dania-Ogbe et al. [30], who suggested that the most significant global threat to biodiversity is the erosion of ethnobotanical knowledge caused by the demise of the aging custodians of this knowledge. This situation is worsened by the weak linkages between domain of science and indigenous knowledge [31].

### 2.2. Gender and Marital Status of Respondents

**Figure 3:**
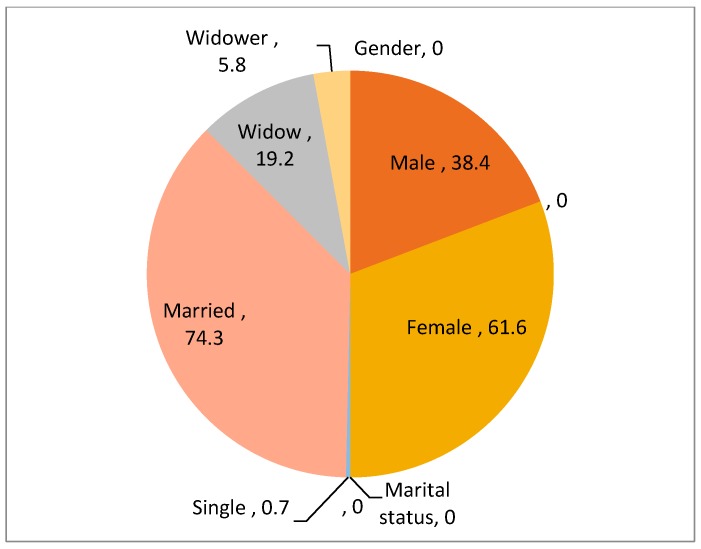
Gender and marital status of respondents.

A gender disaggregation of the respondents in this study showed that the ratio of female to male involvement in the cultivation of and transactions of AYB was approximately 2:1, 61.6% to 34.8%. A higher proportion (74.3%) of the respondents involved in AYB cultivation and processing were married women while the proportions of single individuals, widows and widowers were, respectively, 0.7%, 19.2%, and 5.8% (Figure 3). The dominance of the female gender in AYB cultivation and indigenous knowledge is not unique to Nigeria, as similar findings were reported for the Baka tribe in Cameroon [32] and the Masai in Kenya [33] for knowledge associated with the sales of indigenous plants. The results are also in agreement with the report by Yahaya [34] who reported that 77.9% of married smallholder farmers compared to 22.1% single women were involved in rice farming in Awe Local Government Area of Nasarawa State in Nigeria. The high proportion of female gender was viewed by one of the respondents as follows: “*Africa Yam Bean is regarded as minor and women’s crop; not worthy to engage the energy and labours of the men. They have the patience/endurance of its rigorous attentions and hard-to-maintain practices*”. AYB also serves as a supplementary source of income for female farmers. The cultural characteristics of the study area, where there is often a wide age gap between partners, is exacerbated by the fact that the majority of the respondents lived in rural areas. This provides some reasoning for the dominance of women upon whose shoulders the responsibility of meeting household food requirements usually falls after their older male counterparts are no longer able to undertake manual labour.

### 2.3. Visit of Extension Officers, Size of Farm Land and Sources of Income

**Figure 4:**
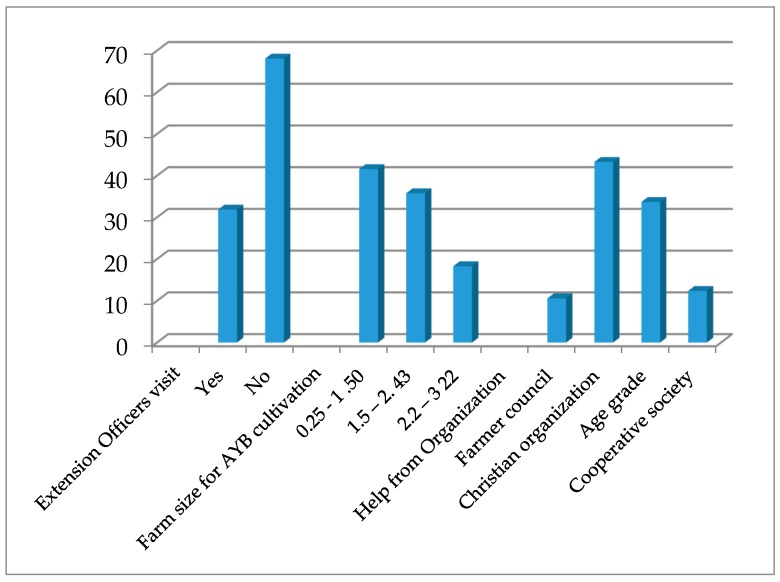
Extension officer’s visits, size of farmland and source of help for farming activities for respondents in the five states of southeastern, Nigeria.

The majority (68%) of the respondents did not receive information or advice from extension officers while 32% claimed to have had contact with them. Similarly, 11%, 43%, 34% and 12% of the respondents normally receive social grants from a farmers’ council, a Christian organization, age grade or a cooperative society, respectively (Figure 4). Aremu et al. [35], noted that the knowledge and application of extension education principles helped extension workers to determine farmers’ needs, constraints, priorities and opportunities connected to their farming activities. They concluded that it also gave them the opportunity to teach farmers the value of improved agricultural practices; recommending suitable crops for different agro-ecological zones and encouraging the adoption of appropriate technologies. The low level of farmer-extension contacts in this study could be implicated as a major contributory factor to AYB being an underutilized crop.

The result also indicated that 41.6% of the respondents had a farm size of less than 0.25–1.50 hectares, 35.9% had between 1.51–2.43 hectares while 18.3% had about 2.2–3.22 hectares (Figure 4). Only 1.9% had a farm size that was more than four hectares. These observations are in agreement with the findings of Nze and Eboh [36] who reported that 70% of respondents in the three agricultural zones of Enugu State had access to ≤3 ha farm land. This could also be attributed to the land tenure system of ownership where women do not have access to land ownership in southeastern Nigeria.

Famoriyo [37] noted that under the customary rules of land tenure, each individual member of a landholding family was entitled to a portion of land, enough to feed himself and the members of ‘his family’, suggesting that men owned land and they apportion it as deemed fit to women. On the contrary, in the study with the Amawbia community in Awka South L.G.A. of Anambra State, Osita [38] noted that farmland allocated to women for cultivation was to keep them busy and to enable them to feed their households from their farm products, and to enable them to cushion the effects of poverty and food insecurity.

### 2.4. Local Nomenclature of African Yam Bean

In southeastern Nigeria, AYB goes by a multiplicity of names based on locality and dialect. At Ngwu Uzuakoli community in Bende LGA of Abia State, AYB is called “Odudu” while in Umuahia North Local Government Area in Abia State it is known as “Akidi”. However, in Anambra and Imo States, it is known as “Okpodudu” while in Enugu State, there are an array of local names among many towns. It is known as “Uzoaki” in Awgu, Aninri, Nkanu North and East Local Government Areas while in Nsukka, Udeni, Igboeze South Igboeze North, and Igbo-Etiti South AYB is called “Ijiriji”. Extending towards the southern part of southeast, Nigeria, to Ebonyi State, African yam bean is called “Uzoaki” in Afikpo and Ohaozara areas while in Izzi, Ikwo and Ohaukwu LGAs it is known as “Azama”. These multiplicities of names were based on the status and or position of this crop in the trado-cultural settings of some of the local communities. It is a food prepared for the labourers when they are hired to work on farm. They eat the food in the morning and will keep on drinking water without getting famished for a very long time. In an idiomatic way, they refer to the crop as ‘6 to 6’, meaning that when you eat AYB by 6 am, while working, you will not need to eat again till 6 pm. AYB is also a crop that sustains the people when other crops are scarce or are all cultivated on the farm. 

### 2.5. Diversity in Seed Coat Colour and Pattern of the AYB Accessions Collected from Southeastern Nigeria

**Figure 5:**
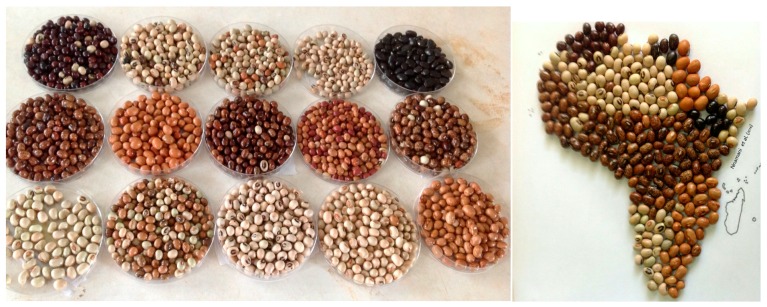
Diversity in seed coat colours and patterns in *Sphenostylis stenocarpa* (*e*x. A. Rich.) Harms. (African yam bean) accessions from five states in southeast, Nigeria. Photo by Nnamani.

A wide variation in seed coat colours and patterns in AYB accessions was observed in this study. The existence of this natural variation across these accessions is very obvious. Colour variation ranges from very light brown to completely black, variegated brown with black shading to black variegated. Others were milky with black eye; milky with brown eye and black (Figure 5). These variations could prove to be useful to both farmers and conservation biologists in providing simple identification of genetic variation, its storage and future development within potential breeding programmes.

FAO [39] noted that traditional varieties have higher stability to adapt to climate variability, change and low-input agriculture under marginal environments thereby facilitating a higher level of resilience for farmers in facing food production risks. This diversity is paramount for varietal breeding as noted by [40]. They concluded that when breeders need to develop new crops with desirable characteristics such as yield potential, greater seed quality, pest and disease resistance, preference is given the species with diverse’ genetic traits, which are of immense importance to farmers and conservation biologists. Seed coat patterning also appears to determine the choice of selection of seeds for cultivation and cooking. Although there were diverse and mixed views about the seed coat pattern, a higher percentage of the respondents preferred the variegated black and brown seed colour to the milk and white seeds (Figure 5). This was based on their assumption that the black and brown variegated seeds were, in their opinion, more proteinous and yielded more when grown. This is in line with the report of Ikhajiagbe and Mensah [41] who noted that the yield of black seeds of AYB per hectare was significantly greater (1542 kg ha^−1^) compared to either the brown variant (1304 kg ha^−1^), or the milky variant (1259 kg ha^−1^). The wide range of diversity inherent in AYB could have contributed to the continuous availability of the crop despite a general level of neglect and limited exploitation in Africa [13].

### 2.6. Food Utilization of African Yam Bean

The corresponding multiple uses of AYB recorded in most of communities is a feature of the cultural diversity in these agro-ecological zones. The array of menus for which AYB is used for food in southeastern Nigeria was wide for a homogenous, linguistically and culturally-knitted group of respondents. The majority of the respondents have good knowledge of this species as part of their diet. However, the older informants (64.3%) were more knowledgeable in this than the younger ones (23%) while 12.7% had no knowledge of other uses of AYB except as snack. This could be attributed to preference of exotic foods to indigenous foods and lack of interest by younger generation.

**Figure 6:**
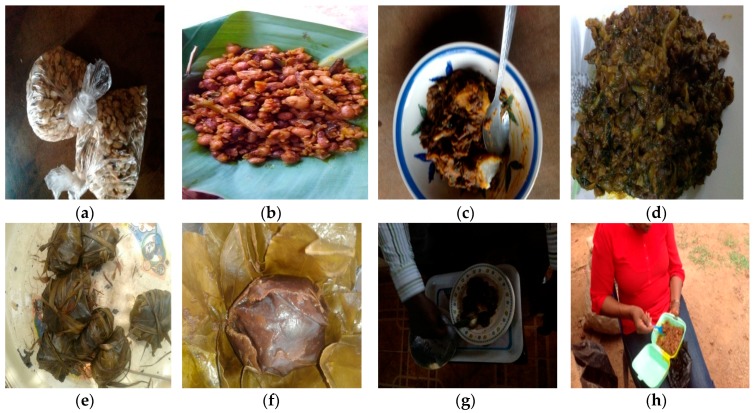
Diversity of foods prepared from *Sphenostylis stenocarpa* accessions in the five states of southeast Nigeria. (**a**–**h**): AYB in association with other crops eaten in southeast Nigeria. (**a**) Fried AYB and eating with palm kneel as a snack; (**b**) Food prepared from AYB with dried cock-yam and “Akpaka—oil-bean seed (*Pentaclethra macrophylla*); (**c**) AYB with Yam; (**d**) AYB cooked as pottage; (**e**–**f**) AYB prepared as mio-mio cake called Ugbagidi in Agwu LGA in Enugu State; (**g**) AYB prepared with ground maize (Ayaraya Oka) and Akpaka; (**h**) Researcher enjoying herself with the delicacy of AYB at Ubani market in Abia State. Source: 2016 Field Survey. Photo © Nnamani.

In Abia State, it is roasted and eaten as snack (Figure 6a) or cooked as pottage, or mixed with “Ugba” (*Pentaclethra macrophylla*), “Okporoko” (stock fish) and served as delicacy at festive events such as traditional marriage, the new yam festival and burial ceremonies (Figure 6b). This is one of the most cherished foods given to a visitor in some communities of Abia State.

Among the communities in Anambra State it is cooked with yam (Figure 6c–d) and served as pottage or made into flour and fried as balls. In Ebonyi State it is roasted or eaten as snack with palm kernel or cooked with yam or as thickener/condiment for soup. Respondents in Enugu State reported that AYB is made into flour and used to prepare moin-moin (Figure 6e–f) or cooked as pottage mixed with vegetables, *Pentaclethra macrophylla*, dried fish (Figure 6d–h) and served as delicacy on festive events such as traditional marriage and naming ceremonies. It is also roasted and eaten as snack with soft palm kernel. It could be cooked with yam and served as pottage or made into flour and mixed with maize flour to prepare foo-foo, and served with “okra” (lady’s finger) soup.

These observations corroborate with the report by Naskashima et al. [42] who noted that indigenous communities, in China, Bolivia and Kenya, favoured the cultivation of varieties of traditional crops over a single high-yielding but also high-risk, mono-cropping system in rural settings. The discovery of these diverse menus in which AYB is prominent in southeastern Nigeria is consistent with the report by [43] that AYB in Ghana, is used extensively in various dietary preparations and this has potentially supplemented the protein requirements of many families throughout the year. They observed that the maintenance of diverse traditional crop varieties and access to seeds were essential tools for the adaptation and survival of poor rural farmers. This enables them to conserve germplasm and provide a contingency when conditions are not favourable.

However, it was observed from the respondents in these zones that they were not aware of AYB having tubers underground which they could equally use.

### 2.7. Income Extractable from the Sales of AYB in the Five States of Southeastern Nigeria

**Figure 7:**
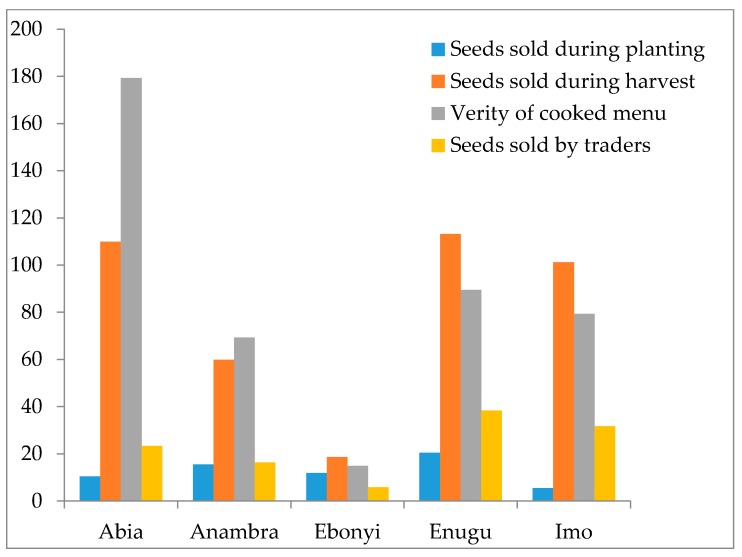
Income extractable from the sales of AYB in the five states of southeastern Nigeria (USD).

Figure 7 shows a summary of the average income generated from the sales of products, seeds and the diversity of menus derivable from AYB. The study showed that the highest income of N16,000 ($180 USD) was generated, particularly in Abia State, from the sales of prepared food of diverse menus from AYB, sold in the open markets, and from road hawkers (Figure 7). The least income was recorded from Ebonyi on the sales of AYB seeds and other food menus. Thus, AYB provides a good complementary source of income for the resource-poor rural and semi–rural dwellers in southeastern Nigeria. This confirms the opinion of Legwaila et al. [44] that food security and poverty alleviation in rural communities can be improved by diversifying the existing few staple crops to include underutilized plants. They concluded that the few staple crops exploited in dry conditions of Botswana could not improve the lives of resource-poor rural households without incorporating alternatives such as indigenous food plants.

Furthermore, experience has shown that many African rural communities actually rely on indigenous plants for food security and as source of cash income between cropping seasons. It has been established that neglected and underutilized species in southeastern Nigeria were potential sources of high levels of essential nutrients which contributed to the daily requirement and maintenance of good health of the resource-poor rural households. It has a direct impact on alleviating poverty, building a sustainable future for these people who are dependent on it as source of livelihood [45].

### 2.8. Other Uses of African Yam Bean in the Five States of Southeastern Nigeria

In all the states, AYB was utilized for many other livelihood services. Respondents enumerated situations where the integration of AYB was crucial for the wellbeing of the people as summarized in Table 1. The ability of the indigenous communities to integrate and interact with their natural surroundings has been a positive approach in their resilience, survival and sustainable development. The utilitarian value of AYB in the communities examined spanned the five states studied. Such utilitarian value includes the use of dried AYB snacks by diabetic patients, extract of mashed cooked AYB to induce lactation after childbirth and the use of the fried ground seed coat to treat strokes (Table 1). This conforms with the expectation implied in the ‘Convention on the Protection and Promotion of the Diversity of Cultural Expressions’ that the recognition of the links between biological and cultural diversity is often embedded with acknowledging the importance of indigenous knowledge and local peoples’ participatory roles in protecting and conserving biodiversity. This is because cultural diversity is a rich asset for safeguarding the vitality of societies; preserving cultural customs and practices and know-how that should be conserved [46].

### 2.9. Constraints to Cultivation and Utilization of AYB

**Figure 8:**
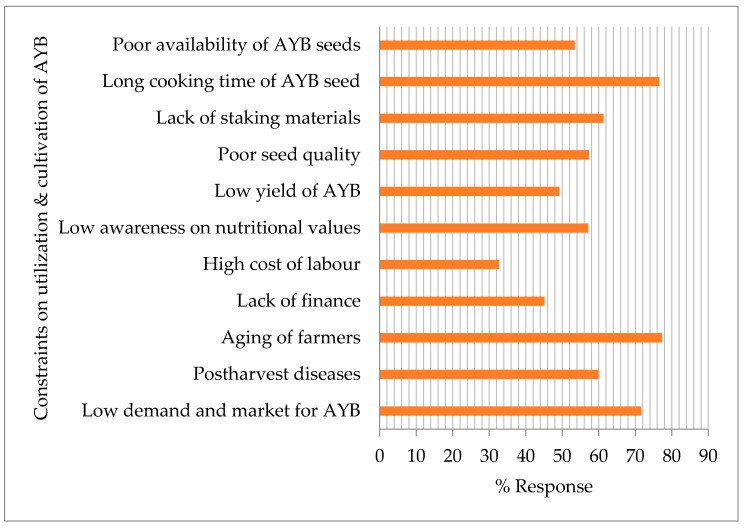
Constraints on the utilization and cultivation of AYB in southeastern Nigeria as determined by data from the 2016 field survey.

AYB utilization and production in southeastern Nigeria is beset with a plethora of problems. Specifically, the challenges confronting its use and cultivation, determined for this study, are summarized in Figure 8. Many of the respondents can be classed as aging farmers (77.3%), and it was suggested that the long cooking time (76.6%) and low product market demand (71.6%) were the most intractable problems confronting AYB cultivation and utilization in southeastern Nigeria. Other constraints identified were the lack of staking materials (61.3%), postharvest diseases (59.9%) and poor awareness on the nutritional values of AYB (57.1%), an important staple as well as a crop of immense cultural value in southeastern Nigeria. Among the strategies for shortening the long cooking time was soaking seeds overnight before cooking and/or adding the petioles of paw-paw (*Carica papaya*) to AYB seeds while cooking to reduce the challenge of long cooking time. These challenges could be attributed to weak linkage between research and indigenous knowledge.

### 2.10. Conservation and Management

**Figure 9:**
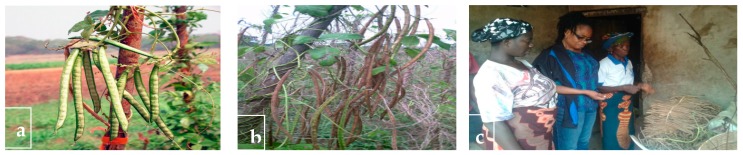
*Sphenostylis stenocarpa.* (**a**) Mature but not ready to harvest the fruits of the African yam bean; (**b**) dried fruits; (**c**) dried harvested fruits stored beside the house by one of the respondents. Photo © Adewale and Nnamani.

Indigenous communities in the study area depend on plant genetic resources for livelihood sustenance. They have developed selective conservation methods to protect these plants including AYB. Conservation of AYB is accomplished through the collection of dried fruits at maturity when the green fruits become brownish within the months of December to February. This is usually after harvesting the yams (*Dioscorea* spp.) with which AYB was staked with in the farm. The most common in situ conservation techniques used were restrictions on use (eating just a fraction of the entire seeds harvested), hanging the dried fruits beside the cooking fire ether inside the kitchen or outside, beside the house and or storing the seeds in calabash gourds, earthenware, plastic cans and bottles. Some of the respondents just tie up the dried fruits in bundles and place them on any dry platform outside the house (Figure 9).

All these conservation methods often expose the seeds to pathogens thereby making them vulnerable to spoilage. This is in line with the reports by the UNESCO [46] that seeds of AYB were usually heavily infected with quite a number of pathogens. With respect to this, considerable efforts, programs and policies should be made to aid the conservation of AYB seeds in the study zones. This is in line with the report of [47] that, to conserve underutilized species effectively, holistic approaches which include both the ex-situ and in-situ techniques must be planned to complement the local conservation strategies.

The remaining seeds of the 34 accessions of AYB used in this project were conserved ex-situ in the germplasm conservation unit of the Biotechnology Research and Development Centre, Ebonyi State University Abakaliki, Nigeria and germplasm screening laboratory, Department of Crop Production and Protection, Faculty of Agriculture, Obafemi Awolowo University, Ile-Ife, Nigeria.

## 3. Conclusions and Recommendations

Globally it is recognized that indigenous knowledge and its institutional systems provide the foundation for participatory strategies for eco-friendly and societally sustainable development. This survey on the voice of the custodians of AYB in this zone has actually contributed to the wealth of knowledge about AYB and its sustaining potential in food security resilience and development.

The social characteristics of respondents involved in AYB activities indicated that the preponderance of the elderly, who are fast aging with little or no formal education, was high. While these traditional custodians of AYB are passing away due to old age, the younger generation has not shown enough interest in this crop.

Furthermore, there was high variability in seed coat colours and patterns and this has a direct relationship with choice of selection of AYB seeds for cultivation and use. However, inadequate information from extension officers, a lack of staking materials, long cooking time, low market demand, lack of appropriate conservation measures and unstable sources of income were among the factors against the cultivation and use of AYB.

Apparently, the results from this study showed that this rich indigenous knowledge could provide a guiding light for informed scientific inquiry to address current challenges. Protection of indigenous knowledge and its promotion among the scientific domain is urgently needed to conserve and develop this crop [48]. As an integral component of the menu of the majority of the agrarian communities in this zone, it could play a significant role on food security and economic development.

This study has unveiled some baseline data on the extensive dietary preparations and other latent uses of AYB in addressing food security and sustainable development of these resource-poor rural communities. Urgent local and national in-situ and ex-situ conservation approaches are highly needed to ensure the continuous existence of this highly promising fading plant genetic resource in the face of change. The National Research Council [49] stressed the need for preliminary surveys, which could quickly be converted into advisory services to farmers who are the keepers of Africa’s age-old yam bean heritage. This, they noted, could be done throughout the AYB zones with the aim of creating more awareness of its multipurpose assets, conserving the germplasm and improving the potentials of this crop.

## 4. Materials and Methods

### 4.1. Study Area

**Figure 10:**
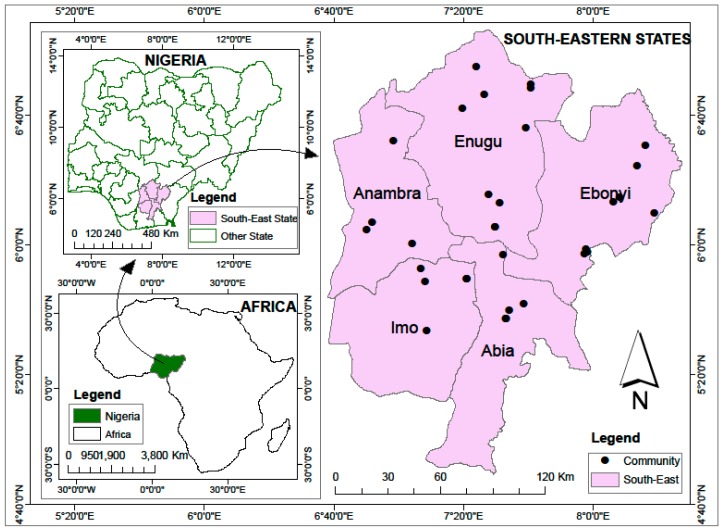
Data collection sites for *Sphenostylis stenocarpa* in southeast Nigeria.

The study area was the southeastern geo-political zone of Nigeria, comprising of Abia, Anambra, Ebonyi, Enugu and Imo states regarded as the Igbo land. It is located within longitudes 5°30′ and 9°30′ E and latitudes 4°30′ and 7°00′ N, occupying a land area of about 75,488 km^2^ (Figure 10) and bordered by the Cross River State to the east, Akwa Ibom to the south, Edo and Delta States to the west, and Kogi and Benue States to the north [50]. Temperature in the study area is characterized by two distinct alternating seasons of uniformly high temperature of 37 °C. The aridity of the dry season is accentuated by the dust-laden harmattan winds (Northeast (NE) Trades Winds). The mean monthly temperatures oscillate between 23.3 °C and 27.7 °C with a seasonal bimodal annual rainfall of 1500–2500 mm. With respect to vegetation, the age-long anthropogenic activities have given rise to a derived mosaic of lowland rainforest vegetation type that houses a relic of the tropical rainforest vegetation belt [50].

### 4.2. Sample Size

Representative samples of 10 respondents were selected randomly from 10 Local Government Areas (LGA) in a state, giving a total number of 500 respondents across the five states (Figure 10). The respondents included community heads, farmers, drivers and vendors of AYB in these communities. The selected 500 respondents were restricted to those were above 18 years of age and older. The 34 accessions collected from the study areas after the research were conserved ex-situ in the germplasm conservation unit of the Biotechnology Research and Development Centre, Ebonyi State University Abakaliki, Nigeria and the germplasm screening laboratory of the Department of Crop Production and Protection, Faculty of Agriculture, Obafemi Awolowo University, Ile-Ife, Nigeria.

### 4.3. Seed/Plant Collection

Verbal pre-informed consent was obtained from the participants before they were interviewed. Interviews were conducted in the local language using guided semi-structured questionnaires and research assistants who were conversant with the local languages. These questionnaires were structured in line with the specific objectives of the study and were administered in the form of an oral interview scheduled in order to ensure that responses to the questions are correctly filled.

The interviews were structured and covered questions pertaining to the uses of AYB, production and utilization constraints. The research questions were focused on (i) identifying the socio-economic status of the respondents; (ii) determining the uses, variability in seed coat colour and patterns; (iii) identifying the effects of climate change including those constraints limiting the use and production system of AYB; (iv) also requested was their knowledge of climate change effect on crops (with particular emphasis on AYB); (v) other uses such as its nutritional, medicinal, and cultural values including income extractable from AYB; and (vi) the demand profile of AYB in these communities. Rapid rural appraisal (RRA) and focal group discussion (FGD) were used to elicit farmers and stakeholders’ awareness and knowledge about climate change, its impact on their major staples; status of AYB and the challenges of cultivating it, while semi-structured interview schedules were used to collect quantitative information from the selected respondents.

Farmers of AYB responded to a five-point scale survey employed to determine the magnitude of their responses: to a very great extent, five points; to a great extent, four points; to some extent, three points; to a little extent, two points; and to a very little extent, one point.

### 4.4. Income Extraction Validation

The nutritional, medicinal, and cultural values, including income and demand profile of AYB in these communities were accessed. Various vendors and different actors on AYB within the local markets participated in this study. The prices for this crop in the various zones as accruing from various transactions were sourced and recorded. Economic evaluation of the plant and its multipurpose uses were inventoried, rating these, and then converting these prices from the local currency to its USA dollar equivalent.

### 4.5. Data Analysis

Data obtained from the questionnaires were processed into a data matrix, percentages and analysed. Data were analysed using simple averages, mean scores and standard deviations with Statistical Analysis System (SAS) to realize the objectives.

## Figures and Tables

**Table 1:** Other uses of African Yam Bean in the five states of southeastern Nigeria.

	Abia	Anambra	Ebonyi	Enugu	Imo
Fodder and part used	-	-	Yes (dried plant after harvesting seed)	Yes (dried plant after harvesting seed)	-
Medicine	Dried YAB snacks are recommended for diabetic patients. It makes them feel full.	Dried YAB snacks are recommended for diabetic patients. It makes them feel full.	Dried YAB snacks are recommended for diabetic patients. It makes them feel full.	Dried YAB snacks are recommended for diabetic patients. It makes them feel full.	Dried YAB snacks are recommended for diabetic patients. It makes them feel full.
	-	Eating AYB induces sleep as a result of its relaxing ability (Insomnia)			Eating AYB induces sleep as a result of its relaxing ability (Insomnia)
	Fried and ground seed is used to treat stroke.	-	-	Fried and grinded seed is used to treat stroke.	-
	Extract of mashed AYB after cooking is used to induce lactation in mothers after birth.		Extract of mashed AYB after cocking is used to induce lactation in mothers after birth	Extract of mashed AYB after cocking is used to induce lactation in mothers after birth. The seed coat of roasted AYB is used in the treatment of stroke by a traditional healer in Ehandiagu found in Nsukka LGA (Clem Uroko).	
Cultural	Used as special food during festivals	Used as special food during festivals	Used as special food during festivals	Used as special food during festivals	Used as special food during festivals
	Women crop	Women crop	Women crop	Women’s crop	Women crop
	Poorer people crop	Poorer people crop	Poorer people crop	Poorer people crop	Poorer people crop

Source: Field survey 2016.
